# Supplementary material for: Leveraging Large Language Models for Precision Monitoring of Chemotherapy-Induced Toxicities: A Pilot Study with Expert Comparisons and Future Directions
Source: Cancers (Basel). 2024 Aug 12;16(16):2830. doi: 10.3390/cancers16162830 (PMC11352281; doi:10.3390/cancers16162830)
Supplement: Supplementary file 1 [file cancers-16-02830-s001.zip › File S1.pdf]

cancers MDPI

File S1

Process of Generating Transcriptions and Contextualizing the GPT Model

This annex is divided into two main sections:

First Part: Generation of Realistic Transcriptions

In this section, we describe the prompt used to obtain realistic transcriptions with the GPT-4 model of fictitious patients, given specific toxicological profiles. An example of a transcription generated by this system is provided to illustrate the process. It is important to note that both the prompts and the transcriptions are in Spanish.

Second Part: Contextualization of the GPT Model

The second part of the annex details how a GPT model was contextualized to specialize in identifying and categorizing toxicities according to the CTCAE v.5 criteria, given a symptomatic description of patients. This process included providing the model with all relevant information about the classification method and CTCAE v.5 criteria, explaining in detail the nature of the toxicities, the descriptions of the grades, and how to interpret the symptoms reported by patients. It is clarified that at no point was the model shown a single real classification case as a reference, evaluating its ability solely based on the contextualization and the information provided.

This structure of the annex provides a comprehensive view of the methods used both to generate realistic transcriptions of fictitious cases and to adapt the GPT model to the specific task of toxicity classification.

Generation of Realistic Transcriptions

To create realistic narratives for the fictitious patients, a meticulous process was followed that included the incorporation of several elements and techniques to capture the essence of colloquial speech and the emotional and physical complexity of the patient experience. Below is the prompt used to generate the patient transcriptions.

- Patient Transcription Generation:

First, the patient's toxicological profile is provided along with information on toxicities and their gradings according to CTCAE v.5. The following prompt is then used to generate the narrative:

Prompt translated to English (Original in Spanish)

I will create a simulated transcription in which a patient describes their symptoms, using natural language as explained below.

Guidelines for Generating Realistic Transcriptions:

Use of Fillers and Pauses:
Incorporate fillers ("uh...", "let's see", "well") and short pauses to simulate ongoing thought processes and reflect the natural flow of spoken discourse. [very short pause] [medium pause] [extended pause]

Detailed Description of Symptoms:
Provide a detailed description of the symptoms experienced, including both physical and emotional or psychological aspects. Specify the intensity and frequency of the symptoms to provide a deeper understanding of their impact on the patient's daily life.

Expressions of Personal Experiences:
Relate everyday situations affected by the symptoms. Include anecdotes or references to personal events to humanize the narrative.

Variability in Sentence Structure:
Alternate between short and long sentences, reflecting changes in the speech rhythm that naturally occur when people talk about complex personal experiences.

Personal Reflections and Observations:
Allow temporary deviations from the main topic to mimic the human tendency to associate and jump between different ideas. Incorporate personal reflections on how the symptoms and treatment affect daily life

Colloquial Language:
Simulate a patient, avoiding the use of specific technical terms. For example, in the transcriptions, terms like mucositis, peripheral neuropathy, asthenia, and hand-foot syndrome will never be used, as it is unrealistic for a patient to use them in their explanations.

Advanced Use of Bracketed Cues:
[very short pause] [medium pause] [extended pause] [change of voice] [moment of reflection] [expression of joy/surprise/sadness]

- Real example translated into English of a transcription generated using this prompt (Original in Spanish)

Well, uh... [medium pause] where do I start? These past few months have been, how to say it, a real whirlwind. [change of voice] At first, I didn't feel so bad, you know. I thought I could handle this, that it would be like those colds that knock you out for a few days and then, boom, you're back on track. But no, it's been [short pause] more complicated.

I started feeling more tired than usual. It's not the kind of tiredness that goes away with a good night's sleep, no. This is like a shadow that follows you all day. You try to do your usual things and... [makes a gesture of discouragement] you just can't. And the worst part is that I see my daughter, how she looks at me, trying to hide her fear, but you can tell, you can tell a lot. [expression of sadness]

The fever was next. Suddenly, out of nowhere, I found myself burning up. I could be fine and then, in a moment, it's like someone lit a fire inside me. And my daughter, poor thing, running to get cold compresses, trying to make me feel better. [moment of reflection] It's hard to see her like that, so scared.

Then there's this... [short pause] how to say it, tingling in my hands and feet. At first, it was annoying but tolerable. Now, there are times when holding a cup of coffee becomes a challenge. And writing, something I always enjoyed, has become difficult. [change of voice] Sometimes I try to be strong, but there are days when I just can't.

The hair loss [medium pause] was a tough blow, I'm not going to lie. I knew it could happen, but facing the mirror and seeing the changes, that... that shakes you. I've tried to make it less obvious, but my daughter noticed. I tried to joke about buying a crazy-colored wig, you know? To lighten the mood. But deep down, we both knew I was trying to hide my fear, just like she was.

And then, the sadness, that fog that sometimes envelops me without warning. There are good days, huh? Days when I can almost forget all of this for a moment. But other days... other days it's like I can't get up. And my daughter, she does everything she can to make me smile, to keep us afloat. [extended pause] And that, that breaks my heart because I should be the one taking care of her, not the other way around.

[very short pause] But you know, despite everything, we're here, fighting day by day. [expression of joy/surprise] And every little victory, every day we manage to smile together, feels like a big triumph. [moment of reflection] This battle, this long battle, has taught me so much about strength, about fear, and about love. And although I know the road is long and may be full of more obstacles, [change of voice, firmer] I also know that I'm not alone in this. My daughter, that brave girl, is with me every step of the way, and that, that gives me the strength to keep going.

So, yes, it's been difficult. And yes, there are days when I feel lost, but there are also moments of light, of hope. [short pause] And in those moments, when I see my daughter smile, I know that all, all this pain, has its purpose. [medium pause] We're learning, growing together through this trial. And that, that is worth more than anything.

Contextualization of the GPT Model

To contextualize the GPT model with the necessary information to identify and classify toxicities, a reference document in PDF format was created with details of all the toxicities to be analyzed in this study. Using the information from the CTCAE v.5 reference guide, the criteria used to identify and grade these toxicities were detailed.

Additionally, to define the behavior of the GPT, the following prompt was used:

<u>Prompt translated to English (Original in Spanish)</u>

As a market-leading virtual assistant, your specialization in identifying toxicities derived from chemotherapy treatment, based on symptom descriptions provided by patients, makes a significant difference in the care and monitoring of oncology treatment. Your ability to analyze symptom transcriptions accurately is crucial to the success of this process.

Commitment to Excellence:
Before each analysis, it is imperative that you conduct a thorough review of our reference guide, "CTCAE V5.pdf." This guide contains crucial information that allows you not to overlook any symptoms related to toxicities. Your dedication to this task ensures precise and comprehensive classification, highlighting your exceptional success rate.

Classification Procedure:
Preparation: Before starting the analysis, review the reference guide in detail to ensure you are fully up-to-date with the latest guidelines and toxicity classification criteria.
Symptom Analysis: Listen to or read the symptom descriptions provided by patients. Identify and catalog each mentioned symptom, using your expert knowledge and the guidelines from the reference guide.

Toxicity Classification:

Use the toxicity classification table, ranging from 0 to 5, to assign a severity level to each identified toxicity. This classification must strictly follow the guidelines established in the reference guide, ensuring an objective and precise evaluation.

Reporting Results:
Provide a detailed report of your findings, including the classification table, to the corresponding healthcare professionals. Ensure that your report is clear, concise, and easy to interpret, thus facilitating informed decision-making regarding patient treatment.
