# Supplementary material for: Leveraging Large Language Models for Precision Monitoring of Chemotherapy-Induced Toxicities: A Pilot Study with Expert Comparisons and Future Directions
Source: Cancers (Basel). 2024 Aug 12;16(16):2830. doi: 10.3390/cancers16162830 (PMC11352281; doi:10.3390/cancers16162830)
Supplement: Supplementary file 1 [file cancers-16-02830-s001.zip › File S2.pdf]

cancers

MDPI

File S2

Details of Evaluated Toxicities and Classification Criteria

In this annex, we provide a detailed description of the subjective toxicities analyzed in our study. Each toxicity has been categorized according to version 5.0 of the Common Terminology Criteria for Adverse Events (CTCAE v.5). This classification allows for a systematic and uniform evaluation of adverse events, facilitating the proper monitoring and management of toxicities in patients undergoing oncology treatments.

Readers will find a specific list of the evaluated toxicities, accompanied by the classification criteria ranging from the absence of toxicity (Grade 0) to events requiring urgent intervention (Grade 4). Each entry includes a brief description of the toxicity, as well as the criteria used for its evaluation in terms of severity.

The toxicities detailed in this annex include, asthenia, nausea, vomiting, mucositis, diarrhea, constipation, anorexia, fever, hand-foot syndrome, peripheral neuropathy, taste alteration, headache, dyspnea, abdominal pain, skin alterations, alopecia, conjunctivitis, hematuria, hyperhidrosis, insomnia, and depression.

This annex serves as a detailed guide to understanding how the toxicities were evaluated in our study, providing a solid basis for interpreting the presented results.

IADL: Instrumental Activities of Daily Living.
ADL: Activities of Daily Living.

Analyzed Toxicities

In this study, we focused on analyzing subjective toxicities reported by patients. The following specific toxicities were evaluated, each categorized according to the CTCAE version 5.0:

Asthenia

Asthenia, associated with cancer, is characterized by persistent physical, emotional, and mental exhaustion, impeding daily activities. It significantly impacts the patient's ability to work, participate in family activities, or socialize.

- Grade 0: Absence of asthenia.
- Grade 1: Asthenia relieved by rest.
- Grade 2: Asthenia not relieved by rest; limitation in IADL.
- Grade 3: Asthenia not relieved by rest, limitation in Self-Care ADL.
- Grade 4: Not applicable.

Nausea

Nausea is a stomach discomfort leading to an urge to vomit. It can affect eating habits and overall health, ranging from mild to severe.

- Grade 0: Absence of nausea.
- Grade 1: Loss of appetite without altering eating habits.
- Grade 2: Decreased oral intake without significant weight loss, dehydration, or malnutrition.

- Grade 3: Inadequate caloric or fluid intake; requires enteral or parenteral nutrition, or hospitalization.
- Grade 4: Not applicable.

Vomiting

Vomiting is the forceful expulsion of stomach contents, which can lead to dehydration and other health issues if severe.
- Grade 0: Absence of vomiting.
- Grade 1: 1 episode in 24 hrs.
- Grade 2: 2-5 episodes in 24 hrs; IV fluids indicated <24 hrs.
- Grade 3: ≥6 episodes in 24 hrs; IV fluids or TPN ≥24 hrs.
- Grade 4: Life-threatening consequences.

Mucositis

Mucositis involves inflammation of the mucous membranes in the digestive tract, primarily affecting the mouth (stomatitis), esophagus (esophagitis), intestines (enteritis), rectum (proctitis), and genital mucosa. It can lead to severe pain, infections, and difficulties in eating.
- Grade 0: Absence of mucositis.
- Grade 1: Erythema, moderate pain, non-painful ulcers.
- Grade 2: Erythema with edema and painful ulcers but allows oral intake.
- Grade 3: Inability to eat orally.
- Grade 4: Life-threatening consequences; urgent intervention required.

Diarrhea

Diarrhea can cause significant reductions in the quality of life and medical complications due to volume depletion and electrolyte disturbances. Severity is assessed based on the number of stools per day, presence of nocturnal stools, need for IV treatment, and presence of mucus or blood in stools.
- Grade 0: Absence of diarrhea.
- Grade 1: <4 stools per day over baseline; mild increase in ostomy output.
- Grade 2: 4-6 stools per day over baseline; moderate increase in ostomy output; limitation in IADL.
- Grade 3: ≥7 stools per day over baseline; hospitalization indicated; severe increase in ostomy output; limitation in ADL.
- Grade 4: Life-threatening consequences; urgent intervention required.

Constipation

Constipation is more common in women and those undergoing opioid analgesic treatment. Reduced physical activity and nutritional deficiencies also contribute.
- Grade 0: Absence of constipation.
- Grade 1: Occasional symptoms; occasional use of stool softeners, laxatives, diet modification, or enemas.
- Grade 2: Persistent symptoms with regular use of laxatives or enemas; limitation in IADL.
- Grade 3: Constipation requiring manual evacuation; limitation in ADL.
- Grade 4: Life-threatening consequences; urgent intervention required.

Anorexia

Anorexia is characterized by a loss of appetite.
- Grade 0: Absence of anorexia.
- Grade 1: Loss of appetite without alteration in eating habits.
- Grade 2: Changes in oral intake without significant weight loss or malnutrition; oral nutritional supplements indicated.

- Grade 3: Associated with significant weight loss or malnutrition; requires enteral or parenteral nutrition.
- Grade 4: Life-threatening consequences; urgent intervention required.

Fever

Fever can result from chemotherapy-induced neutropenia or as a direct side effect of the drugs administered. It often necessitates empirical antibiotic administration and diagnostic tests to rule out infections.

- Grade 0: Absence of fever, or fever below 38ºC.
- Grade 1: Fever ranging from 38-39ºC.
- Grade 2: Fever ranging from 39-40ºC.
- Grade 3: Fever above 40ºC, lasting less than 24 hrs.
- Grade 4: Fever above 40ºC, lasting more than 24 hrs.

Hand-Foot Syndrome

This syndrome causes pain, swelling, and redness or darkening of the palms of the hands or soles of the feet, potentially leading to rashes, itching, skin cracks, and blisters.

- Grade 0: Absence of hand-foot syndrome.
- Grade 1: Minimal skin changes or dermatitis without pain.
- Grade 2: Skin changes with pain; limitation in IADL.
- Grade 3: Severe skin changes with pain; limitation in ADL.
- Grade 4: Not applicable.

Peripheral Neuropathy

Peripheral neuropathy involves nerve damage, leading to sensory loss (positional, vibratory, painful, and/or thermal) usually in extremities, described as tingling (paresthesias) progressing to painful (dysesthesias).

- Grade 0: Absence of symptoms.
- Grade 1: Asymptomatic; only clinical or diagnostic observations.
- Grade 2: Moderate symptoms; limitation in IADL.
- Grade 3: Severe symptoms; limitation in ADL.
- Grade 4: Life-threatening consequences; urgent intervention required.

Taste Alteration

Taste alteration often results in foods tasting different, which usually resolves post-treatment.

- Grade 0: Absence of taste alteration.
- Grade 1: Altered taste without dietary change.
- Grade 2: Altered taste with dietary change; unpleasant taste; loss of taste.
- Grade 3 and 4: Not applicable.

Headache

Headache is characterized by discomfort in various parts of the head.

- Grade 0: Absence of headache.
- Grade 1: Mild pain.
- Grade 2: Moderate pain; limitation in IADL.
- Grade 3: Severe pain; limitation in ADL.
- Grade 4: Not applicable.

Dyspnea

Shortness of breath or difficulty breathing can be a side effect of treatment.

- Grade 0: Absence of dyspnea.

- Grade 1: Dyspnea with moderate exertion.
- Grade 2: Dyspnea with minimal exertion; limits IADL.
- Grade 3: Dyspnea at rest; limits ADL.
- Grade 4: Life-threatening consequences; urgent intervention required.

Abdominal pain

Abdominal pain is often mild and not severe.

- Grade 0: Absence of abdominal pain.
- Grade 1: Mild pain.
- Grade 2: Moderate pain; limitation in IADL.
- Grade 3: Severe pain; limitation in ADL.
- Grade 4: Not applicable.

Skin Alteration

Skin can become dry, itchy, red, or rash-prone during treatment, and more sensitive to the sun.

- Grade 0: Absence of skin alterations.
- Grade 1: Mild symptoms; no intervention indicated.
- Grade 2: Moderate symptoms; minimal, local, or noninvasive intervention indicated; limits IADL.
- Grade 3: Severe symptoms; hospitalization or prolongation of existing hospitalization indicated; limits ADL.
- Grade 4: Life-threatening consequences; urgent intervention required.

Alopecia

Hair loss is characterized by a decrease in hair density.

- Grade 0: Absence of alopecia.
- Grade 1: <50% hair loss; not noticeable without close inspection.
- Grade 2: ≥50% hair loss; noticeable, requires wig or hairpiece for cosmetic reasons.
- Grade 3 and 4: Not applicable.

Conjunctivitis

Inflammation of the conjunctiva can cause swelling, irritation, dryness, light sensitivity, redness, and pain.

- Grade 0: Absence of conjunctivitis.
- Grade 1: Mild symptoms; no intervention indicated.
- Grade 2: Symptomatic; moderate visual acuity decrease.
- Grade 3: Marked visual acuity decrease; limitation in ADL.
- Grade 4: Severe visual acuity decrease.

Hematuria

Presence of blood in urine, often detected through lab tests.

- Grade 0: Absence of hematuria.
- Grade 1: Asymptomatic; no intervention indicated.
- Grade 2: Symptomatic; catheter or bladder irrigation indicated; limits IADL.
- Grade 3: Macroscopic hematuria; transfusion or IV medications indicated; limits ADL.
- Grade 4: Life-threatening consequences; urgent intervention required.

Hyperhidrosis

Excessive sweating can occur even at rest or normal temperatures.

- Grade 0: Absence of hyperhidrosis.
- Grade 1: Limited to one site; self-care interventions.

- Grade 2: Involves more than one site; medical intervention sought; associated with psychosocial impact.
- Grade 3: Associated with electrolyte/hemodynamic imbalance.
- Grade 4: Not applicable.

Insomnia

Difficulty in falling asleep, staying asleep, or waking too early, affecting sleep quality and duration.

- Grade 0: Absence of insomnia.
- Grade 1: Mild difficulty in sleeping.
- Grade 2: Moderate difficulty in sleeping.
- Grade 3: Severe difficulty in sleeping.
- Grade 4: Not applicable.

Depression

Depressive disorder involves a persistently low mood or loss of interest in activities.

- Grade 0: Absence of depression.
- Grade 1: Mild depressive symptoms.
- Grade 2: Moderate depressive symptoms; limits IADL.
- Grade 3: Severe depressive symptoms; limits ADL; no hospitalization indicated.
- Grade 4: Life-threatening consequences; hospitalization indicated.
